# Quantifying the Effect of Colony Size and Food Distribution on Harvester Ant Foraging

**DOI:** 10.1371/journal.pone.0039427

**Published:** 2012-07-10

**Authors:** Tatiana P. Flanagan, Kenneth Letendre, William R. Burnside, G. Matthew Fricke, Melanie E. Moses

**Affiliations:** 1 Department of Biology, University of New Mexico, Albuquerque, New Mexico, United States of America; 2 Department of Computer Science, University of New Mexico, Albuquerque, New Mexico, United States of America; 3 The Santa Fe Institute, Santa Fe, New Mexico, United States of America; University of Arizona, United States of America

## Abstract

Desert seed-harvester ants, genus *Pogonomyrmex,* are central place foragers that search for resources collectively. We quantify how seed harvesters exploit the spatial distribution of seeds to improve their rate of seed collection. We find that foraging rates are significantly influenced by the clumpiness of experimental seed baits. Colonies collected seeds from larger piles faster than randomly distributed seeds. We developed a method to compare foraging rates on clumped versus random seeds across three *Pogonomyrmex* species that differ substantially in forager population size. The increase in foraging rate when food was clumped in larger piles was indistinguishable across the three species, suggesting that species with larger colonies are no better than species with smaller colonies at collecting clumped seeds. These findings contradict the theoretical expectation that larger groups are more efficient at exploiting clumped resources, thus contributing to our understanding of the importance of the spatial distribution of food sources and colony size for communication and organization in social insects.

## Introduction

According to optimal foraging theory, an animal’s foraging behavior should maximize its net energy intake and minimize its costs [1], [2]. The distribution of food affects how animals forage; many forage in groups when food is clumped and individually when food is more dispersed [3]–[6]. Group foraging ranges from the passive and uncoordinated, as when solitary foragers happen to congregate at scarce food patches, to actively coordinated collection of food that either requires cooperation (e.g. wolves preying on elk), or whose collection benefits from communication among group members.

Ant foraging behaviors vary among species, ranging from largely independent foraging to mass recruitment. Foraging behaviors are affected by distribution of food [7], [8] and colony size [3], [9]. We use a field study to quantify how the distribution of food affects foraging rates in seed harvesting ants. We also examine how the size of the forager population affects the relationship between food distribution and foraging rate.

### Food Distribution

Foraging behavior of ants can be affected by the relative richness of the food source [7], [10]–[15]. Studies have shown that granivorous ants prefer to collect seeds from dense piles [7], [16]. Davidson [12] found that foraging activity was correlated with periods of high seed density. Other studies have found that this preference is also correlated with preference for particular seeds [7], [17], but when the abundance of preferred seeds diminishes, ants will forage on the more abundant species [7]. Denny [17] suggests that some ants concentrate their foraging efforts on relatively clumped food, presumably because of the greater energetic returns on their foraging investments. For example, the argentine ant *Iridomyrmex humilis* lays pheromone trails, recruiting other ants to newly discovered food sources [18]. New World leafcutter ants (*Atta* and *Acromyrmex* spp.) create large visible trunk trails in order to harvest massive quantities of leaves clumped on individual trees [19]. The seed harvester ants *Pogonomyrmex occidentalis*, *P. barbatus* and *P. rugosus* can exhibit strong recruitment responses in which nestmates are led to dense seed piles [18], [20]–[22].

The spatial distribution of seeds consumed by *Pogonomyrmex* ranges from highly clumped to randomly dispersed. Reichman [23] found extreme variability in the density of seeds collected by these ants in the Sonoran Desert, with a 78-fold difference in seed density across space, including a 25-fold difference within microhabitats. Edeleman [24] found three-fold increases of seeds surrounding kangaroo rat mounds in the Chihuahuan desert. Thus, we expect desert seed harvesters to have foraging strategies that are effective given highly variable distributions of seeds.

### Forager Population

Ant colonies range in size from dozens to millions of workers [3]. Foragers from smaller colonies are less likely to communicate to coordinate foraging, while larger colonies tend to exhibit a high degree of worker cooperation [9]. Smaller colonies spend more time searching, with less success than the group foragers of large colonies [23]. Beckers et al. [3] show more integration through sophisticated communication networks as colonies increase in size; some of them based on permanent trail-laying behavior [25]. The ability of ant species with large colonies to efficiently harvest dense food sources is in part a result of these communication strategies [3], [19], [25], [26]. Large networks of foragers linked by chemical communication may allow large colonies to exploit food more efficiently because they enable more effective group decisions or because the local availability of more individuals allows the mobilization of a larger, faster response [9], [18].

Harvester ants vary substantially in total colony population size. Forager population size varies substantially between species, between colonies of the same species, and over time for any particular colony [8]. Johnson [27] estimated total colony population sizes in the Chihuahuan Desert of New Mexico as thousands for *P. rugosus*, a few hundred for *P. Maricopa,* and fewer than 100 for *P. desertorum* . Whitford and Ettershank [28] estimated colony forager populations of dozens in *P. desertorum* and thousands in *P. rugosus.* Since not all members of the colony actively forage, total colony population is an upper bound on forager population size.

### Pogonomyrmex Foraging

*Pogonomyrmex* are relatively large-bodied ants found mostly in arid regions of South, Central, and North America [29], where the distribution of seeds is expected to be heterogeneous [10]. They are well studied, monodomous central-place foragers whose primary diet is local seeds found on the top layer of soil [3], [23], [26], [27]. Although all harvester ants consume seeds and multiple species often occur in sympatry (with colonies of several species often found within a few meters of each other), individual species differ in average body size, the size of seeds eaten, and average mature colony size [12], [14].

Although *Pogonomyrmex* individuals communicate and coordinate tasks in their underground nests, it is not clear whether and how foragers use communication networks or pheromone trails during foraging. They are able to use pheromone trails to recruit foragers to large piles of seeds, as when supplied experimentally [30], but it is not clear that they commonly use pheromone recruitment to piles of food under natural conditions. While prior studies found evidence of recruitment [22], [31], [32], some found that foraging is regulated by other behaviors [8], [33]. For example, site fidelity is a foraging strategy in which individual workers return to search for food in the same general place or direction where it was found before [34], [35]. *Pogonomyrmex* foragers exhibit strong site fidelity, returning to search for food in the same general direction they successfully foraged [32]. A more recent study found that *P. barbatus* foragers return to the location where they last found a seed [36].

Site fidelity can reduce the time an ant spends foraging. Foraging time can be seen as the composition of two distinct activities. When a forager leaves its nest in search of food, it travels in a general direction, presumably established by a pheromone trail or by directional fidelity [37]. The time it takes for an ant to arrive at a general area where it starts to search for food, is the *travel time*. Once at the destination, the ant engages in a more localized search. The time it takes to find a patch of food in this general area is the *search time.* Once a patch of food is discovered, each ant that returns to that pile takes the approximate same travel time, but its search time is reduced. Beverly et al. [36] showed that search time is a much larger component on total trip duration than travel time.

### Approach

We expect that seeds can be collected significantly faster when they are clumped in large piles because the search component of foraging time is reduced once ants have discovered the location of a pile of seeds. If ants communicate the locations of seed piles, the discovery of a pile by one forager would reduce the search time of other foragers. We expected harvester ant foragers to preferentially harvest densely piled seeds, maximizing their foraging efficiency by minimizing their search time.

We studied colonies from three sympatric *Pogonomyrmex* species that vary substantially in forager population size and asked how forager population size affects foraging rates for seeds in different spatial distributions. In turn, we posit that the foraging patterns we observed reflect underlying behavioral mechanisms. Understanding how foraging rates depend on food distribution and colony size may guide future studies of behavioral strategies in these and other ants.

Due to natural variation in colony size and food distribution, *Pogonomyrmex* seed harvesters are ideal for testing hypotheses about how food distribution and forager population size favor different foraging strategies. In this study, we test whether seed harvester colonies exploit large dense seed piles faster than small dispersed piles, and if so, how much faster. To test how the clumpiness of seed distributions affects foraging, we measure foraging rates as the number of seeds collected per unit time, on seeds clumped into piles of different sizes. We use a procedure to normalize our measures of foraging rates to account for variations in colony size and experimental food distribution. We compare the foraging rates on piled seeds to the foraging rates on randomly distributed seeds for each experiment. We also test whether forager population size affects the rate of foraging on piled versus randomly distributed seeds.

## Materials and Methods

We focused on three sympatric species of desert seed-harvester ants in the genus *Pogonomyrmex* in the high desert of central New Mexico: *P. desertorum, P. maricopa* and *P. rugosus*. We carried out this fieldwork in the summer of 2008 and 2009 in a mid-succession lot of approximately 13 hectares in Albuquerque, New Mexico, in the Chihuahuan desert of the southwestern U.S. No permissions were required to access the locations of these activities, and no permits were required for the described field studies.

To establish a quantitative relationship between the distribution of seeds and foraging rates for colonies that varied in forager population size, we used two key variables: We estimated average forager population size of each species and experimentally manipulated the distribution of seeds. We use repeated measures ANOVA to measure how forager population size and the distribution of seeds affected the rate at which seeds were collected.

### Estimating Active Forager Population Size

Our initial estimates of forager population size were based on observations from an earlier study [38] that were carried out in the summers of 2003 and 2004, where we tracked 63 individual ants from 13 colonies in the McKenzie Flats area of the Sevilleta Long Term Ecological Research site in central New Mexico. Observations of these colonies provided preliminary estimates for each of the three species of average forager population size, average distance traveled to collect a seed, and average time to collect a seed.

Individual foragers were followed as they left the nest, traveled to a search location, searched for and acquired a seed, and returned to the nest. We marked some foragers either with paint (DecoColor opaque paint Uchida of America) or colored chalk powder; others were followed and left unmarked. For each forager we measured the time to complete a foraging trip (*T_f_*) from nest to seed and back to the nest, the linear distance from nest to seed (*d_s_*) and travel velocity (*v_t_*) of each forager returning with the seed to the nest. The measurements are reported in Table 1.

**Table 1:** Means* for characteristic variables of each species estimated at the Sevilleta LTER in 2003–2004.

Variable/Spp	N	Mean	SE	Lower Bound	Upper Bound
***Tf***	Total time of round trip from the time an ant leaves the nest to the time it returns with a seed [minutes]				
*P. desertorum*	15	7.25	2.73	1.84	12.67
*P. maricopa*	16	17.96	2.85	12.29	23.62
*P. rugosus*	32	13.56	2.24	9.11	18.01
***dt***	Measured distance between seed and nest [meters]				
*P. desertorum*	15	3.86	1.04	1.8	5.92
*P. maricopa*	16	8.18	1.09	6.02	10.34
*P. rugosus*	32	7.83	0.85	6.14	9.53
***vt***	Travel velocity. Calculted as dt/return time [meters/minute]				
*P. desertorum*	15	2.96	0.49	1.97	3.95
*P. maricopa*	16	2.25	0.48	1.29	3.21
*P. rugosus*	32	3.10	0.34	2.42	3.77
***R***	Rate of foragers returning to the nest measured at equilibrium [foragers/minute]				
*P. desertorum*	15	8.73	3.08	2.61	14.86
*P. maricopa*	16	12.50	2.99	6.57	18.43
*P. rugosus*	32	107.94	2.11	103.74	112.13
***F***	Number of active foragers (calculated from foraging trip time multiplied by mean seed intake rate)				
*P. desertorum*	15	77.88	196.49	−312.37	468.13
*P. maricopa*	16	208.52	190.25	−169.34	568.38
*P. rugosus*	32	1712.61	134.53	1445.43	1979.8

* Based on modified population marginal means. N is the number of experiments conducted to measure each variable.

We estimated the number of active foragers by multiplying the average time of a foraging trip (*T_f_*) by the rate that ants return to (and leave from) the nest when the rates of leaving and returning ants were at equilibrium. At equilibrium the number of ants that are foraging (*F*) is constant. The rate of ants leaving the nest at equilibrium (*R_eq_*, which is equal to the rate ants return to the nest) multiplied by *T_f_* provides an estimate of the active forager population (*F*) at a particular time: *F* = *T_f_* *R_eq_. We estimated the number of foragers per species at the in 2004 at the Sevilleta LTER using this method. We calculated the equilibrium rate by counting each ant leaving the nest for three minutes and each ant returning for 3 minutes. When these numbers differed by less than 10 percent, we considered that an equilibrium flux of ants (Table 1). We multiplied that number by average foraging trip time (*T_f_*) to get the active forager population (*F*) for that colony for that day (Table 1). We used these initial estimates of *F* to design our field study, and we repeated these methods to estimate *F* for the same species at the site in which our 2008 and 2009 seed manipulation studies were conducted.

### Manipulative Seed Studies

To measure the effect of seed dispersion on foraging rates, we conducted manipulative field experiments on 21 colonies of three *Pogonomyrmex* species in the summers of 2008 and 2009. We conducted a total of 37 replicates of a field experiment on eight colonies of *P. desertorum,* four colonies of *P. maricopa* and nine colonies of *P. rugosus.*

**Figure 1:**
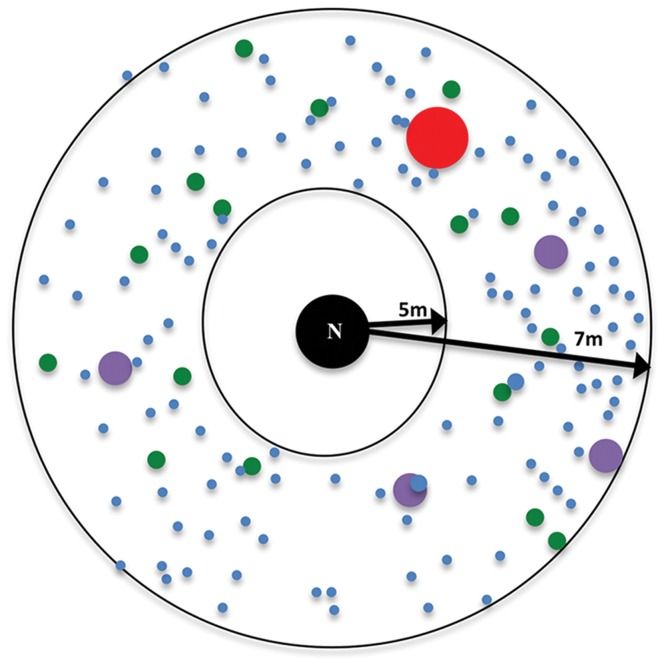
Experimental seed distribution around the nest entrance of a *P. rugosus* colony. Each colored circle is a pile of millet seeds dyed to that color. The size of each circle represents the relative number of seeds in that pile: red = 1-pile of 256 seeds, purple = 4-piles of 64 seeds, green = 16-piles of 16 seeds, and blue = 256 individual seeds distributed randomly.

We began observations each morning to coincide with the start of daily foraging activity. We selected an active colony and baited it with dyed seeds arranged in a wide ring around the colony entrance (Figure 1). Seeds were dyed with Americolor Soft Gel Paste food coloring.

We placed dyed seeds in four distributions of different colors, equal in number but varying in degree of dispersion: one pile of red seeds, four piles of purple seeds, sixteen piles of green seeds and a random scattering of blue seeds. Piles of seeds of all colors were dispersed at the same average distance for all colonies of the same species. For *P. rugosus* the average distance was 6 meters (seeds were placed between 5 and 7 meters from the nest). For *P. maricopa* and *P. desertorum* the average distance was 3 meters (seeds were placed between 2 and 4 meters from the nest). See Table 2 and Figure 1. Regardless of the pile size, we distributed the seeds in every pile evenly over a 10×10 cm^2^ area. The start time for each experiment was marked when the first seed was placed. After placing the experimental baits, an observer recorded the color of each seed brought into the nest with a time stamp using a computer program we created. We concluded observations either when a focal colony ceased foraging or when ants had collected all experimental baits, usually 60–90 minutes after the start-time of the observation.

**Table 2:** Experimental seed distribution.

Species	Distance from nest	Distribution	Color	Number of seeds per pile
*P. desertorum*	2–4 m	1 pile	Red	32
(8 colonies)		4 piles	Purple	8
		16 piles	Green	2
		Random	Blue	1
		*Total*		128
*P. maricopa*	2–4 m	1 pile	Red	32
(4 colonies)		4 piles	Purple	8
		16 piles	Green	2
		Random	Blue	1
		*Total*		128
*P. rugosus*	5–7 m	1 pile	Red	256
(9 colonies)		4 piles	Purple	64
		16 piles	Green	16
		Random	Blue	1
		*Total*		1024

**Figure 2:**
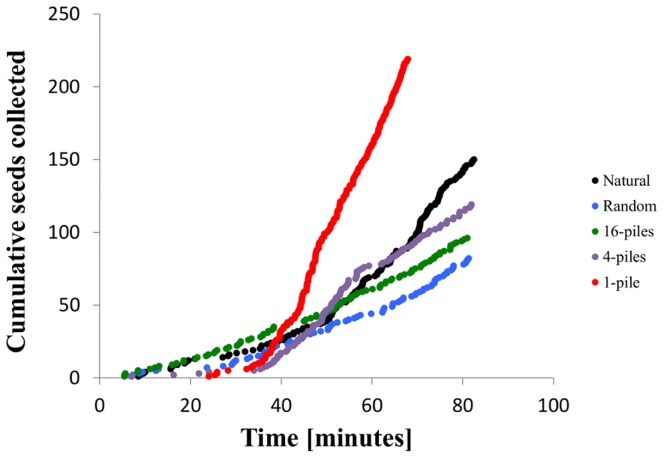
Seed intake rates from one field observation of a colony of *P. Rugosus*. The y-axis shows the cumulative number of seeds collected by the time specified on the x axis. Red = 1 pile of 256 seeds, purple = 4 piles of 64 seeds, green = 16 piles of 16 seeds, blue = 256 piles of 1 seed (randomly scattered seeds).

This method produced five time series from each experiment, one each for seeds from each experimental seed distribution and one for unmanipulated naturally occurring seeds. An example set of four cumulative seed collection curves from one observation is shown in Figure 2.

### Accounting for Variation in Colony Size and Conditions

The three species in our study vary widely in average forager population size, territory size (Table 1) and also in their ability to handle seeds. Further, daily variation in colony activity means that the active forager population size varies substantially for the same colony [8]. Thus, there is large variation between species, across colonies within a species, and through time for a particular colony.

We dealt with variation in colony size in two ways. First, we dealt with systematic species differences by varying the experimental treatment given to each colony. Second, we used a procedure to normalize our measures of foraging rates to account for both the differences in experimental treatment and fluctuations in the number of active foragers.

Experimental treatments varied between species so as not to disadvantage small colonies. By placing fewer seeds closer to smaller colonies with smaller average foraging distances, we attempted to provide an equal chance for an individual forager to encounter one of our experimental seeds independent of whether that individual was in a large or small colony (Table 1). Based on the forager population estimates for each species from studies in 2003 and 2004, we chose a number of seeds roughly proportional to the forager population size: 1024 seeds for *P. rugosus* and 128 seeds each for *P. desertorum* and *P. maricopa* (which were on average approximately 8 times smaller than *P. rugosus* colonies). To obtain a similar density of seed patches on the territories of different species, we adjusted the distances of baits from the nest entrances in each treatment. We placed the baits in a ring 5–7 m from each *P. rugosus* colony, and 2–4 m from each colony of *P. maricopa* and *P. desertorum* (Table 2). Because the distance that a forager typically travels does not increase linearly with the number of foragers in a colony, it was not possible to simultaneously keep the density of seeds constant and the distance from nest to seed precisely proportional to typical forager travel distance.

In preliminary studies we noticed that *P. desertorum* foragers, with the smallest body size, frequently had difficulty handling the hulled millet with which we baited the other species. Because recruitment responses may be reduced with excessive handling times of large grains [39] we baited *P. desertorum* colonies with sesame seeds. All three species readily collected experimental seeds whenever they encountered them, suggesting that seed preference was not a significant factor.

To determine whether seeds clumped in larger piles were collected faster than the same number of seeds dispersed across smaller piles, we calculated the rate (Equation 1) at which seeds are collected from each of the piled distributions (seeds in 1, 4 or 16 piles), and we calculated the same rate for seeds scattered at random.
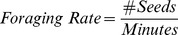
(1)


Because colonies varied widely in the number of foragers, activity and experimental treatments, we used a distribution of randomly scattered bait seeds as a control to normalize our observed foraging rates across experiments. The *normalized foraging rate* describes the rate at which piled seeds are collected *relative to randomly scattered seeds* within each experiment. To calculate the normalized rates, we divided the rate at which each clumped distribution was collected by the rate at which randomly scattered seeds were collected (Equation 2). This allowed us to analyze foraging rates on piled seeds *relative* to foraging rates on random seeds.
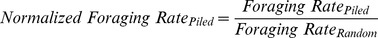
(2)


Equation 2 provides three different normalized foraging rates for each experiment: one for the seeds in a single large pile, one for seeds in four medium piles, and one for seeds in 16 small piles.

The randomly scattered seeds serve as a control for each experiment because all of the following are the same for the random seeds as the piled seeds: the average distance between seeds and the nest, the density of seeds, the type of seed (millet or sesame), and the number of foragers that are active during an experiment. Thus, all variables that are difficult (or impossible) to control across experiments are the same for random and piled seeds within each experiment. When we divide the foraging rate on each piled distribution by the foraging rate on the random distribution, the only parameter that changes is the clumpiness of the distribution. This allows us to compare how changes in seed distribution affect foraging rate across experiments, colonies and species. We emphasize that our methods are *not* designed to answer the question “How fast do foragers collect seeds?” but rather “How fast do foragers collect piled seeds compared to randomly scattered seeds?”

### Data Analysis

We considered foraging in two time periods. The first was defined as the time from the placement of seeds (the start of the experiment) to the time that the first seed from each distribution was collected. This *discovery time* measures the amount of time for an ant to find a seed from each distribution. The second time period was defined as the time from the discovery of the first seed of a distribution to the last seed collected from that distribution. In this second time period we calculated both a *foraging rate* (Equation 1) and a *normalized foraging rate* (Equation 2). The foraging rate shows how fast seeds are collected once an ant has discovered a seed from that distribution. The normalized foraging rate shows how much faster piled seeds are collected compared to randomly scattered seeds. In Table 3 we report, for each species, the discovery times, foraging rates and normalized foraging rates for each species. We focus our analysis on the normalized foraging rates.

**Table 3:** Mean* discovery times.

Species	Distribution	Discovery Times	
		Mean	SE
*P. desertorum N = 9*	Natural	9.578	3.361
	1 pile	25.99	5.842
	4 piles	20.393	5.688
	16 piles	16.571	3.821
	Random	11.551	3.365
*P. maricopa N = 9*	Natural	13.035	3.881
	1 pile	21.942	6.746
	4 piles	27.55	6.568
	16 piles	16.187	4.412
	Random	15.772	3.885
*P. rugosus N = 9*	Natural	9.238	3.229
	1 pile	20.547	5.613
	4 piles	19.829	5.465
	16 piles	9.813	3.671
	Random	11.669	3.233
*Combined species*	Natural	10.617	2.022
	1 pile	22.827	3.514
	4 piles	22.591	3.422
	16 piles	14.190	2.299
	Random	12.998	2.024

* Based on modified population marginal means. Discovery times are calculated as the total time in minutes from the start of the observation to the retrieval of the first seed for each distribution.

To test for trends in foraging rates for the different species and distributions, we analyzed both foraging rates and normalized rates using a repeated measures general linear model [40] with species and distribution as independent variables and foraging rates as a dependent variable. We included species as a between-subject factor and seed distribution as a repeated measure in these analyses.

We additionally tested for effects of forager population size on normalized foraging rates within species. We did not estimate active forager population size for each experiment, so we used the rate at which a colony collected natural seeds in each experiment as a proxy for active colony size during that experiment. Therefore, we tested for a relationship between each of the three normalized foraging rates (Equation 2) and the foraging rate on natural seeds.

## Results

To ensure that our experimental design did not bias results, we conducted two tests. We tested for bias in the collection or observation of bait seeds of different color by observing colonies of each species foraging in piles of bait seeds of mixed colors, with equal numbers of the four colors in a single pile. The test showed no bias by color in the order of arrival of seeds at the focal nests (Kruskal-Wallis test: *n* = 802 seeds; *p* = 0.59). We measured foraging rates of naturally occurring seeds as well as rates for each colored seed distribution (Table 4). The mean foraging rates for natural seeds are similar to those for our baits, indicating that our measured rates are not an artifact of baiting the ants with extraordinary amounts of food.

**Table 4:** Mean* foraging rates and normalized rates for each seed distribution.

Species	Distribution	First seedto Last Seed			Start-timeto Last Seed	
		Mean	SE		Mean	SE
*P. desertorum*	Natural	0.610	1.292		0.569	0.826
N = 9	1 pile	0.683	0.485	N = 15	0.249	0.316
	4 piles	0.316	0.266		0.138	0.190
	16 piles	0.350	0.123		0.173	0.112
	Random	0.314	0.095		0.235	0.136
	*Total*	2.273				
*P. maricopa*	Natural	3.286	1.218		3.025	1.066
N = 9	1 pile	1.018	0.457	N = 9	0.650	0.408
	4 piles	0.842	0.251		0.367	0.245
	16 piles	0.485	0.116		0.383	0.145
	Random	0.429	0.090		0.328	0.175
	*Total*	6.060				
*P. rugosus*	Natural	3.696	1.218		3.591	0.887
N = 9	1 pile	3.436	0.457	N = 13	2.340	0.339
	4 piles	1.726	0.251		1.438	0.204
	16 piles	1.490	0.116		1.345	0.121
	Random	1.005	0.090		1.080	0.146
	*Total*	11.353				
*Measure: Normalized foraging rates*						
*P. desertorum*	Natural	2.555	8.142		4.289	5.858
N = 9	1 pile	2.491	1.352	N = 15	1.325	0.809
	4 piles	1.175	0.426		0.638	0.254
	16 piles	1.177	0.195		0.868	0.171
*P. maricopa*	Natural	17.041	8.142		18.654	7.562
N = 9	1 pile	4.072	1.352	N = 9	3.182	1.045
	4 piles	2.361	0.426		1.392	0.328
	16 piles	1.320	0.195		1.328	0.220
*P. rugosus*	Natural	4.190	8.142		4.102	6.292
N = 9	1 pile	3.614	1.352	N = 13	2.550	0.869
	4 piles	1.790	0.426		1.373	0.273
	16 piles	1.582	0.195		1.494	0.183
*Combined species*	Natural	7.929	4.701		9.015	3.817
N = 27	1 pile	3.392	0.781	N = 37	2.352	0.527
	4 piles	1.775	0.246		1.135	0.166
	16 piles	1.360	0.112		1.230	0.111

* Based on modified population marginal means. Total collection time used to calculate the rates is measured from first seed to last seed of each distribution and from the time the observation starts to last seed of each distribution.

We estimated an active forager population (mean ± standard error) of 71±341 for *P. desertorum*, 269±185 for *P. maricopa*, and 356±211 *P. rugosus* in our 2009 study. The *P. maricopa* and *P. desertorum* estimates are similar to those estimated in earlier years at the Sevilleta NWR (Table 1), but the *P. rugosus* estimates are significantly smaller.

Discovery times were unaffected by species identity (*p*>0.05), but not surprisingly, were longer in the more clumped distributions across all species (*p* = 0.002, Table 3). Averaged over all species, discovery times for random seeds (13.00±2.02 minutes) and seeds in 16 piles (14.19±2.23 minutes) were indistinguishable, and discovery times for seeds clumped in 4 piles (22.59±3.42 minutes) and 1 pile (22.83±3.51 minutes) were indistinguishable. Figure 2 shows the cumulative number of seeds collected over time for each distribution in one field experiment. The x-intercept measures the time it took for each distribution to be found.

**Figure 3:**
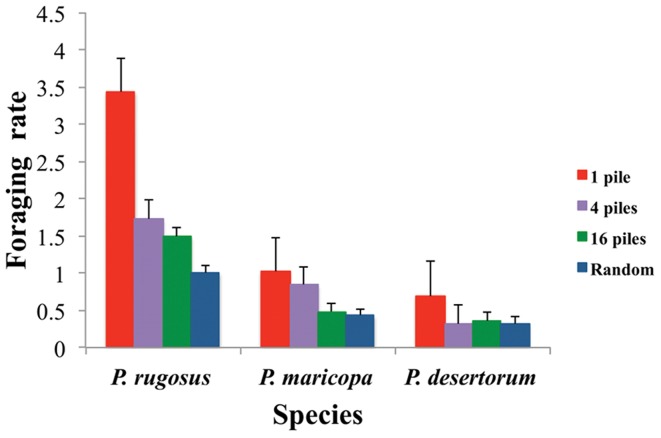
Mean foraging rate. The bars show mean foraging rates on piled seeds. Rates are measured in seeds/minute per species and distribution. Error bars are standard errors.

Foraging rates (seeds collected per minute from the time of arrival of the first seed to the time of arrival of the last seed) are significantly different between species (*p*<0.001), indicating that species with larger colonies have higher absolute foraging rates. The analysis also shows a significant difference in foraging rates between pile sizes within species (*p*<0.001). Within each of the three species, foraging rate decreases as seeds are dispersed across more piles. According to paired t-tests, foraging rates for 4-pile, 16-pile, and random distributions are significantly different from the 1-pile distribution for *P. rugosus* (*p = *0.008, 0.011 and 0.009 respectively) and *P. desertorum (*p = 0.004, 0.025 and 0.012 respectively). Due to high variation, foraging rates are not significantly different between distributions for *P. maricopa*. Foraging rates are shown in Table 4 and Figure 3.

**Figure 4:**
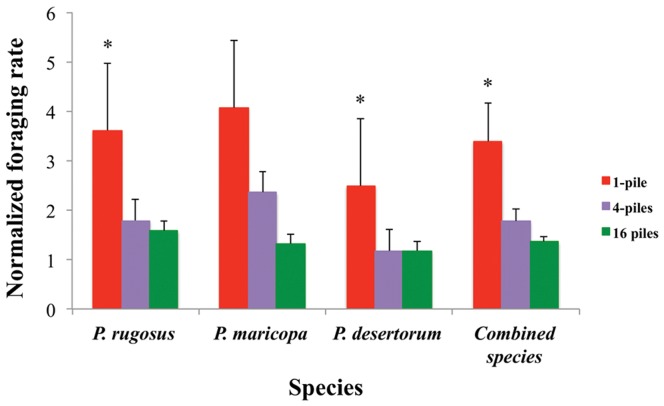
Normalized foraging rates. Bars indicate *normalized rates* (foraging rate on piled seeds divided by foraging rate on random seeds) for three distributions for three species depicted separately and combined. A value of 1 indicates that seeds from a piled distribution are collected at the same rate as randomly distributed seeds. Asterisks indicate significant differences of the single pile distribution rates with all other rates within the same species. Error bars are standard errors.

Once we normalized foraging rates (Figure 4) by dividing the rates for each distribution by the rate of randomly distributed seeds, we found that species had no significant effect on normalized rates (p = 0.07), but seed distribution affected normalized rates within species (*p*<0.024). According to paired t-tests, normalized rates for 4-pile and 16-pile distributions are significantly different from the 1-pile distribution for *P. rugosus* (*p<0.05*). Normalized rates for the 1-pile distribution are significantly different from the 4 pile distribution for *P. desertorum.* As is the case with foraging rates, normalized rates are not significantly different between distributions for *P. maricopa*.

Since we found no significant effect of species on normalized foraging rates, we grouped the data from all three species to compare normalized foraging rates across all experiments. We found that the normalized foraging rates declined significantly from the 1-pile distributions to the more dispersed 4- and 16-pile distributions (*p*<0.05). The marginal means ± standard error of combined normalized rates are 3.4±0.7, 1.8±0.2 and 1.4±0.1 for 1-pile, 4-pile and 16-pile distributions of seeds respectively, which indicates that foraging rates increase as seeds are more clumped. Table 4 summarizes the marginal means for foraging rates and normalized foraging rates for all species and distributions. For each individual species, and all species combined, there is a trend for 4 piles to be collected faster than 16, and for the single pile, there is a large and significant increase in normalized foraging rate.

We additionally tested for evidence of a relationship between active forager population size and normalized rates within species. We used the rate of natural seeds collected for each experiment (varying from 0.44 to 18.35 seeds per minute) as a proxy for forager population size. For each species, we tested whether each of the three normalized rates had a significant correlation with the rate of natural seed collection. In these nine comparisons, the only significant relationship was that that larger *P. maricopa* colonies had a higher normalized foraging rate on the 1-pile distribution (*p*<0.001). The lack of significant relationships between our proxy of colony size and normalized foraging rate in the other eight comparisons again indicates that forager population size does not affect the rate of collection of clumped versus random seeds.

## Discussion

We observed foraging by three sympatric species of *Pogonomyrmex* on experimentally manipulated seed distributions and quantified the effect of seed distribution and forager population size on foraging rate. Not surprisingly, more-clumped distributions were collected faster by all ant species (Figure 4), suggesting that all species reduced foraging times on clumped distributions, minimizing the cost of searching for seeds, supporting previous studies on social foraging [12], [14], [31], [41]. However, given hypothesized differences in foraging strategies in larger colonies reported in previous studies [41] we were surprised to find no evidence that colonies with large forager populations collected clumped seeds relatively faster than smaller colonies.

All ant species systematically increased foraging rates on seeds in more clumped distributions. When we clump the same number of seeds into 16, 4 and 1 pile, normalized foraging rates increased from 1.4 to 1.8 to 3.4. This indicates that seeds in 16 piles are collected 40% faster, seeds in 4 piles are collected 80% faster and seeds in 1 pile are collected 340% faster than randomly scattered seeds. Thus, these ants exploit clumped distributions to collect seeds faster. However, the increase in foraging rate with pile size is sublinear – a pile that is 16 times bigger is collected only 3.4 times faster.

The rate at which ants collect seeds is a function of two processes – the search time, which depends on the time the ants take to discover a seed from a distribution, and the travel time, which is the time it takes to carry seeds from a distribution once it is found. For all species, the time to discover more dispersed (random and 16-pile) seeds was faster than the time to discover more clumped (4- and 1-pile) seeds. However, once those piles were discovered, clumped seeds were collected significantly faster than the dispersed seeds. We analyzed the rate at which ants collected seeds from each piled distribution relative to randomly scattered seeds, and this normalized foraging rate indicated how much faster foraging occurs once a colony knows the location of one seed from a distribution, eliminating the search time from the total foraging time. The normalized foraging rate also accounts for differences in the number of active foragers in a given day and allowed us to make comparisons across variations in species, conditions, and experimental treatment.

Not surprisingly, colonies with more foragers collected a larger total number of seeds (Figure 3). However, repeated measures analysis showed no effect of species (which vary significantly in forager population size; see Table 1) on normalized foraging rates. Because prior work suggests that larger colonies are more likely to use some form of group recruitment, we expected that large colonies might be disproportionately good at collecting seeds from large piles, because of the larger number of foragers available to collect these seeds. However, all colonies collected seeds from large piles faster than seeds from small piles, regardless of species identity or our estimate of colony size within species. This study suggests that large and small colonies of *Pogonomyrmex* allocate relatively similar proportions of foragers to large piles to collect them faster. Figure 4 shows that the increase in foraging rate with pile size is indistinguishable for large and small colonies. It is possible that the lack of an observed colony size effect results from variability in forager population size, and the difficulty of measuring it, within and between species.

Additionally, these results should be interpreted in the context of our study design. We controlled for colony territory size and for the distance that foragers travel to look for food by placing seeds closer to smaller colonies, giving large and small colonies equal opportunity to access the seed piles. However, this resulted in a higher density of piles in the territories of species with small colonies compared to the density of piles for larger colonies. In natural settings, it is possible that large colonies more often exploit large piles because their larger territories contain more large piles.

Our study does not reveal the specific foraging behaviors that these ants employ to collect clumped seeds faster, but we do suggest that two strategies are plausible. Clumped seeds in our study could have been collected faster by some form of recruitment, or as a result of a behavior called site fidelity, or a combination of both behaviors.

Previous work has shown that colonies with large numbers of ants and sophisticated communication networks recruit more effectively [3], [25], [26]. Thus, if the foragers we studied used some form of recruitment, we would have expected larger colonies to collect bigger piles relatively faster (we would have expected a higher normalized foraging rate for larger colonies). We did not observe this. One explanation may be that these harvesters do not use pheromone recruitment. Another possibility is that the ants in our study do use some sort of group recruitment, but allocate only a small number of additional foragers to collect from even very large piles. If large and small colonies each allocate a similar small fraction of foragers to collect from large piles, this could explain why large and small colonies forage equally fast on large piles relative to random seeds.

Some have hypothesized that seed harvesters rarely recruit in nature because seeds are distributed heterogeneously over time rather than over space [41]. Further, *Pogonomyrmex* use a site fidelity behavior, where foragers repeatedly return to the last place that they found a seed [8], [37]. This foraging behavior allows ants to exploit large piles faster because a single ant repeatedly returning to the same pile reduces its search time. Site fidelity may be sufficient to collect piles of seeds quickly [36], [42]. For seed piles small enough that a single ant can collect all the seeds in a patch before the colony ceases foraging activity for the day, there may be no benefit in recruiting other foragers to that pile. If ants primarily use site fidelity and not recruitment, then we would expect large and small colonies to be equally capable of collecting large piles faster, as we saw in our field study. However, in the case of a pile so large that the seeds cannot be collected by a single ant in a foraging period, or when seeds might be taken by competitors if they are not collected rapidly, recruitment of other ants to the site may be much more beneficial.

In other work [43], we use an agent-based model to show that pheromone recruitment results in increased foraging rates on more clumped distributions; however, pheromone recruitment alone results in lower normalized foraging rates than we observed in our field study. Site fidelity may provide an alternative explanation for how these seed harvesters collect large piles faster. In ongoing modeling work we explore how the processes of site fidelity and pheromone recruitment may each contribute to the ants’ exploitation of seeds in different distributions.

While our findings suggest no differences in foraging strategy among these species, this stands in contrast to descriptions of interspecific variation within *Pogonomyrmex* in foraging strategy in the literature e.g. [27]. If larger colonies do not exploit their numbers to collect piled seeds substantially faster than smaller colonies, how do they compensate for the energetic needs of more ants and longer traveling distances? It is possible that smaller colonies are capable of the foraging strategies that allow them to exploit more densely distributed foods when given the opportunity to do so, even though larger colonies more often have opportunity by virtue of their larger territory size, given random placement of patches of food in the environment.

Further studies specifically designed to measure foraging rate given the same distribution of seeds for all colonies are warranted, particularly since native seed distributions are not adjusted so that more small piles occur closer to small colonies. The effect of colony size might be very different given the same distribution of food for all colonies or given competition for food between colonies of different sizes. Since colony size has profound effects on colony life history [44] and foraging strategy [25] this should be a fruitful area for further study.

Our study shows that ants from three *Pogonomyrmex* species systematically increase foraging rates as seeds are clumped into fewer large piles. The species differ substantially in forager population, but the increase in foraging rate when seeds are clumped into larger piles is consistent across all three species. The increase in foraging rate on more dispersed distributions is surprisingly slow–increasing only three- to four-fold when clumped in piles 16 times bigger. Other foraging studies, for example Deneubourg et al. [45] suggest that foragers of heavily recruiting ants converge very quickly on rich resources. This may suggest that seed harvesters, which forage on resources that remain relatively static over the course of a foraging period, spend more time exploring for new seeds rather than exploiting known piles of seeds. Understanding how different species of ants balance the trade-off between exploiting known resources versus exploring for new ones may improve understanding of foraging behavior in other animals that forage collectively.
